# Serum IL-6 concentration is a useful biomarker to predict the efficacy of atezolizumab plus bevacizumab in patients with hepatocellular carcinoma

**DOI:** 10.1007/s00535-024-02185-w

**Published:** 2024-12-09

**Authors:** Ryoichi Miura, Atsushi Ono, Hikaru Nakahara, Yuki Shirane, Kenji Yamaoka, Yasutoshi Fujii, Shinsuke Uchikawa, Hatsue Fujino, Eisuke Murakami, Tomokazu Kawaoka, Daiki Miki, Masataka Tsuge, Takeshi Kishi, Waka Ohishi, Naoya Sakamoto, Koji Arihiro, Clair Nelson Hayes, Shiro Oka

**Affiliations:** 1 Department of Gastroenterology, Graduate School of Biomedical and Health Sciences, Hiroshima University Hospital, Hiroshima, 734-8551 Japan; 2 Department of Clinical Oncology, Graduate School of Biomedical and Health Sciences, Hiroshima University, Hiroshima, Japan; 3 Liver Center, Hiroshima University, Hiroshima, 734-8551 Japan; 4 Biosample Research Center, Radiation Effects Research Foundation, Hiroshima, Japan; 5 Department of Clinical Studies, Radiation Effects Research Foundation, Hiroshima, Japan; 6 Department of Pathology and Clinical Laboratories, National Cancer Center-Hospital East, Kashiwa, Chiba Japan; 7 Department of Anatomical Pathology, Hiroshima University Hospital, Hiroshima, Japan

**Keywords:** Unresectable hepatocellular carcinoma, Atezolizumab plus bevacizumab, Interleukin-6

## Abstract

**Background:**

This study aims to identify biomarkers for treatment response of atezolizumab plus bevacizumab (Atezo+Bev) in patients with hepatocellular carcinoma (HCC).

**Methods:**

96 patients who received Atezo+Bev or lenvatinib as a first-line systemic therapy were enrolled as the training group after propensity score matching (PSM), and 42 patients treated with Atezo+Bev were enrolled as the validation group. 17 serum cytokines were measured by Luminex multiplex assay at the start of treatment. For further assessment of the association between cytokine levels and the tumor microenvironment (TME), immunohistochemistry (IHC) was performed on pre-treatment liver biopsy specimens.

**Results:**

In the derivation set, multivariate analysis identified elevated IL-6 as an independent risk factor in the Atezo+Bev group (HR 5.80: p<0.01), but not in the lenvatinib group; in a subset analysis of patients with low IL-6, PFS was longer in the Atezo+Bev training group than in the lenvatinib group (*p* = 0.02). A validation study also showed a significantly longer prognosis in the low IL-6 group for both PFS (*p* = 0.0001) and OS (*p* = 0.03). Serum IL-6 had a positive correlation with tumor IL-6 expression (*ρ* = 0.56, *p* < 0.0001) and an inverse correlation with the CD8/CD163-positive cell count ratio (*ρ* = −0.4, *p* < 0.01).

**Conclusion:**

Serum IL-6 levels are thought to be involved in the suppression of tumor immunity and are useful in predicting the therapeutic effect of Atezo+Bev treatment.

**Supplementary Information:**

The online version contains supplementary material available at 10.1007/s00535-024-02185-w.

## Introduction

Hepatocellular carcinoma (HCC) is one of the most common malignancies and a leading cause of cancer-related death worldwide [1]. Recently, atezolizumab plus bevacizumab (Atezo+Bev) was approved as a first-line treatment for patients with unresectable HCC (uHCC). However, the efficacy of the treatment varies from case to case; in clinical trials, 30% of patients achieved an objective response, whereas 19% failed to achieve disease control [2, 3]. Therefore, it is desirable to develop relevant biomarkers to identify patients who are most likely to benefit from Atezo+Bev therapy.

Currently, the most reliable method for forecasting efficacy is to evaluate the tumor microenvironment (TME) of the tumor tissue. For example, HCC with high CD274 and T_eff_ signatures or with CD8-positive cell infiltration are considered to have hot TME, and Atezo + Bev are effective [4]. However, because tumor biopsies entail the risks of dissemination and bleeding, liver cancer is one of the cancers in which tissue biopsy is relatively difficult to perform. Therefore, there is a need to discover blood biomarkers that are non-invasive and easily evaluated, and in this study, we focused on cytokines. Cytokines such as IL-6 and IL-8, which have immunomodulatory effects, are known to promote cancer growth in TME [5, 6]. IL-8 has been reported to be a prognostic factor in patients undergoing immune checkpoint inhibitor (ICI) treatment for malignant melanoma, lung cancer, and renal cell carcinoma [7–10], and high levels of IL-6 may be useful in predicting treatment response in HCC treated with Atezo+Bev therapy [5, 6]. However, high serum cytokines such as IL-6 and IL-8 are often associated with malignancies that are resistant to treatment and have a poor prognosis [11–14], and it can be difficult to determine whether levels of these cytokines reflect response to Atezo+Bev treatment or indicate progression of the underlying tumor potential. This study evaluated whether the identified markers were specific to Atezo+Bev by comparing them to patients treated with lenvatinib.

## Methods

Please refer to the Supplementary materials and methods for more detailed descriptions.

### Patients and study design

**Fig. 1:**
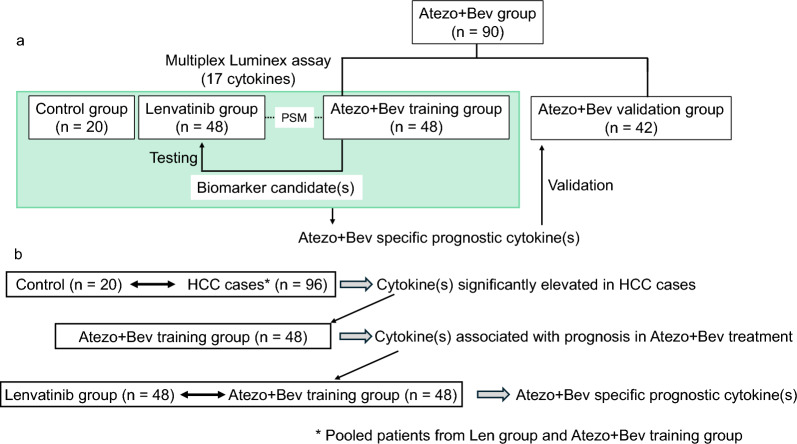
Study design. **a** The Atezo+Bev group was divided into a training group and a validation group. In the Atezo+Bev training group, 17 cytokines were measured using a Luminex assay. Promising markers were tested in cases of Len, matching the backgrounds of the Len and Atezo+Bev training group through propensity score matching. The cytokine measurements utilized the same Luminex plate as that used in the Atezo+Bev training group. If Atezo+Bev-specific prognostic markers were identified, they were subsequently validated in the Atezo+Bev validation group. **b** The process for identifying Atezo+Bev-specific prognostic cytokines

A total of 324 uHCC patients, including 163 patients who started Atezo+Bev therapy between October 2020 and October 2022, and 161 patients who started lenvatinib treatment between April 2018 and October 2022 at Hiroshima University Hospital, were enrolled in this study. Patients were excluded based on the following criteria: were not receiving first-line treatment, were enrolled in a clinical trial involving lenvatinib, had a relative dose intensity (RDI) of less than 75% within 1 month of starting lenvatinib treatment, could not be assessed by imaging, or discontinued lenvatinib within 1 month or discontinued Atezo+Bev within 3 months for reasons other than treatment-related adverse events or efficacy. After applying these criteria, 58 patients treated with lenvatinib and 90 patients treated with Atezo+Bev remained. The background of these 148 patients is shown in Supplementary Table 1, and the study design is shown in Fig.1a. The Atezo+Bev group was divided into a training group (*n* = 48) and a validation group (*n* = 42). A clinically matched Len group (*n* = 48) was also established using propensity score matching with the Atezo+Bev training group. Seventeen cytokines were measured in the Atezo+Bev training group using a multiplex Luminex assay. Promising markers were tested in the lenvatinib group, utilizing the same Luminex plate used for measurements in the Atezo+Bev training group. If Atezo+Bev-specific prognostic markers were identified, they were subsequently validated in the Atezo+Bev validation group. The process for identifying Atezo+Bev-specific prognostic cytokines is shown in Fig. 1b. As a further analysis of the mechanism, immunohistochemistry (IHC) staining for CD163, CD8, and IL-6 was performed on tumor biopsy tissues from Atezo+Bev patients before administration to evaluate the relationship between serum IL-6 levels and the TME of tumor tissues. The target patients were 40 of the 90 patients in the Atezo + Bev group for whom tumor biopsies had been performed, and the patient background is described in Supplementary Table 2. Collection of serum samples for this study was approved by the Hiroshima University Ethics Committee in accordance with the Declaration of Helsinki [15] (approval numbers E-726-2 and HI-98, respectively). All patients provided prior written informed consent for the collection and use of data and samples for the study and publication.

## Results

### Patient background characteristics

Baseline demographics for patients in the background-matched Atezo+Bev training and lenvatinib groups are shown in Supplementary Table 3. Approximately 80% of the patients were male, the median age was 73 years, and 90% of the cases were Child–Pugh class A. 60% of the patients were classified as BCLC stage intermediate. No significant differences were observed in any parameters between the Atezo+Bev training group and the lenvatinib group.

### Comparison of serum cytokine levels between HCC and non-HCC cohort

The background factors of patients in the HCC and non-HCC (control) groups in whom multiplex Luminex assay was conducted are shown in Supplementary Table 4. Chemokine results are presented in Supplementary Table 5 and Supplementary Fig. S2. Of the 12 chemokines compared, IL-6, Angiopoietin-2, IL-8, and CEACAM-1 were significantly higher in the tumor group (IL-6: *p* = 0.02, Angiopoietin-2: *p* = 0.007, IL-8: *p* = 0.003, CEACAM-1: *p* = 0.001). Four cytokines were considered to be tumor-derived factors and were subjected to further analysis.

### Analysis of serum cytokines in the Atezo+Bev training group

**Fig. 2:**
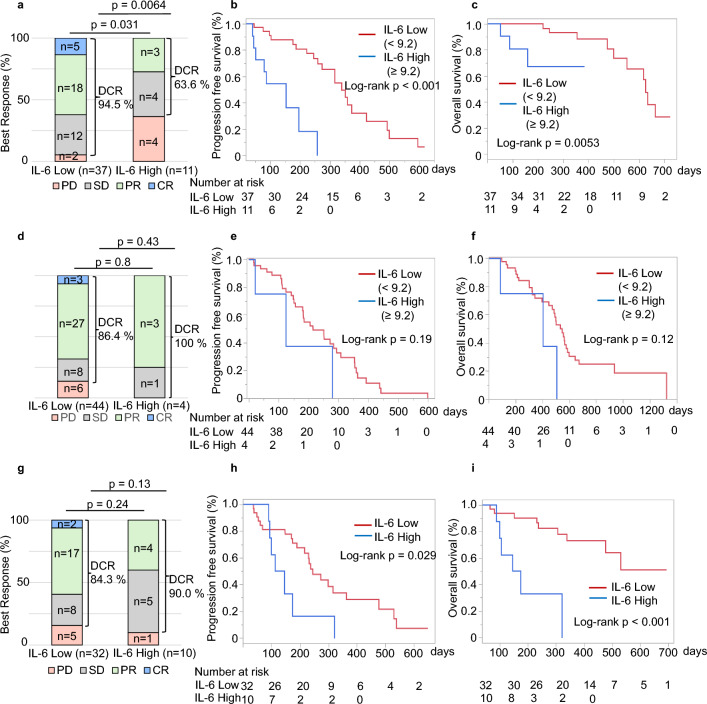
Comparison of therapeutic responses to high- and low-serum IL-6 levels in Atezo+Bev therapy. **a**, **b**, **c** Best response to high- and low-serum IL-6 levels in the Atezo+Bev training group (**a**) and Kaplan–Meier curves for progression-free survival (PFS) (**b**) and overall survival (OS) (**c**). **d**, **e**, **f** Best response to high- and low-serum IL-6 levels in the lenvatinib group (**d**) and Kaplan–Meier curves for PFS (**e**) and OS (**f**). **g**, **h**, **i** Best response to high- and low-serum IL-6 levels in the Atezo+Bev validation group (**g**). and Kaplan–Meier curves for PFS (**h**) and OS (**i**)

To evaluate the prognostic value of Atezo+Bev therapy for uHCC with respect to IL-6, Angiopoietin-2, IL-8, and CEACAM-1, we evaluated their association with PD in the best response. We determined the optimal cutoff for distinguishing between best response as PD and non-PD by plotting a receiver operating characteristic (ROC) curve using Youden’s index. Furthermore, we calculated the q value using FDR (Table 1). To evaluate the efficacy of IL-6, IL-8, and CEACAM-1 as prognostic factors, univariate and multivariate analyses using the Cox proportional hazards model were performed for PFS with the addition of other clinical parameters, including tumor factors (Table 2). Univariate results showed that high IL-6 (HR (95%CI) 7.1 (2.5–19.9), *p* = 0.0002), high IL-8 (HR (95%CI) 3.4 (1.3–8.5), *p* = 0.01), and CRAFITY score ≥2 (HR (95%CI) 5.7 (1.5–21.0), *p* = 0.0097) were associated with shorter PFS, while multivariate analysis revealed that high IL-6 level (HR (95%CI) 5.6 (1.5–21.0), *p* = 0.01) was an independent predictor of prognosis (Table 2). The ORR was 62.2%, and DCR was 94.5%. In terms of disease control, there were significantly more cases in the low IL-6 group (IL-6 High/Low; 63.6/94.5 %, *p* = 0.0064), and both OS and PFS were significantly prolonged in the high IL-6 group (OS, PFS; *p* = 0.0053, *p* < 0.001) (Fig.2a–c).

**Table 1:** Logistic regression analysis of factors related to PD in Atezo+Bev training group

	Cutoff	Odds rate	95% CI	p value	q value
Angiopoietin-2	≥3010.9, 3010.9	3.7	(0.53–25.7)	0.16	0.16
CEACAM-1	≥44703, 44703	5	(0.81–31.0)	0.064	0.087
IL-6	≥9.2, 9.2	0.1	(0.01–0.65)	0.0064	0.026
IL-8	≥102.3, 102.3	0.17	(0.02–1.03)	0.036	0.072

*CEACAM-1* carcinoembryonic antigen-related cell adhesion molecule 1; *IL-6* interleukin-6; *IL-8* interleukin-8

**Table 2:** Cox proportional hazards analysis of factors related to PFS in Atezo+Bev training and lenvatinib group

		Atezo+Bev						Lenvatinib					
		Univariate analysis			Multivariate analysis			Univariate analysis			Multivariate analysis		
		HR	95% CI	p value	HR	95% CI	p value	HR	95% CI	p value	HR	95% CI	p value
Age (years)	≥75/75	1.51	(0.71–3.19)	0.28				0.75	(0.37–1.5)	0.42			
Sex	Male/female	0.49	(0.22–1.11)	0.09				1.11	(0.38–3.2)	0.84			
ECOG PS	0/1 and 2	0.69	(0.28–1.73)	0.43				1.56	(0.66–11.5)	0.66			
Etiology of liver diseases	Viral/non-viral	1.54	(0.73–3.26)	0.26				1.31	(0.67–2.6)	0.42			
Child–Pugh class	B/A	0.5	(0.11–2.21)	0.36				3.92	(1.5–10.2)	0.005	2.66	(0.88–7.96)	0.08
ALBI score	≥−2.36/−2.36	1.35	(0.63–2.88)	0.44				1.29	(0.66–2.50)	0.45			
Tumor diameter (mm)	≥30/30	1.66	(0.77–3.57)	0.19				0.82	(0.41–1.65)	0.59			
Intrahepatic tumor	Single/multiple	1.52	(0.35–6.64)	0.58				1.32	(0.22–2.51)	0.58			
Macrovascular invasion	Yes/no	0.71	(0.27–1.89)	0.49				3.16	(1.3–7.5)	0.009	2.29	(0.84–6.21)	0.1
Extrahepatic spread	Yes/no	2.0	(0.91–4.4)	0.08				0.95	(0.47–1.95)	0.9			
BCLC stage	A and B/C	0.65	(0.31–1.39)	0.27				0.9	(0.46–1.78)	0.77			
AFP (ng/mL)	≥200/200	1.43	(0.65–3.11)	0.37				1.14	(0.57–2.31)	0.7			
DCP (mAU/mL)	≥651.5/651.5	1.19	(0.56–2.54)	0.64				1.07	(0.55–2.07)	0.84			
NLR	≥5.0/5.0	0.84	(0.28–2.50)	0.75				1.4	(0.49–4.0)	0.53			
PLR	≥300/300	0.85	(0.11–6.42)	0.88				3.55	(0.78–16.1)	0.1			
CRAFITY score	2/0 and 1	5.65	(1.52–21.0)	0.0097	2.78	(0.66–11,8)	0.17	2.05	(0.6–7.04)	0.25			
CEACAM-1 (pg/mL)	≥44703/44703	0.93	(0.40–2.17)	0.87				0.51	(0.22–1.17)	0.11			
IL-6 (pg/mL)	≥9.2/9.2	7.1	(2.53–19.9)	0.0002	5.59	(1.49–21.0)	0.01	2.2	(0.66–7.39)	0.2			
IL-8 (pg/mL)	≥102.3/102.3	3.36	(1.33–8.51)	0.018	1.15	(0.32–4.17)	0.83	1.11	(0.51–2.39)	0.79			

*AFP* α-fetoprotein; *ALBI score* albumin and bilirubin score; *Atezo+Bev* atezolizumab plus bevacizumab; *BCLC* Barcelona Clinic Liver Cancer; *CRAFITY score* CRP and AFP in immunotherapy score; *DCP* des-γ-carboxy prothrombin; *ECOG PS* Eastern Cooperative Oncology Group Performance Status; *IL-6* interleukin-6; *IL-8* interleukin-8; *NLR* neutrophil-to-lymphocyte ratio; *PFS* progression-free survival; *PLR* platelet-to-lymphocyte ratio

### Analysis of serum cytokines in the lenvatinib group

There were no significant differences in best response, PFS, and OS between the groups with IL-6 levels above 9.2 pg/mL and those below in lenvatinib group (Fig. 2d, e, f, respectively). To evaluate the prognostic value of lenvatinib therapy for uHCC, we performed the same analyses as for Atezo + Bev with regard to IL-6, angiopoietin-2, IL-8, and CEACAM-1 (Supplementary Table 6). The results of univariate and multivariate analyses using the Cox proportional hazards model for PFS in the lenvatinib validation set are presented in Table 2. Univariate results suggest that Child–Pugh class B (HR (95%CI) 3.92 (1.5–10.2), *p* = 0.005) and MVI positivity (HR (95%CI) 3.16 (1.3–7.5), *p* = 0.009) are associated with shorter PFS, while multivariate analysis revealed that these two items were not independent factors.

### Independent validation of serum IL-6 as a prognostic predictor in Atezo+Bev validation group

A validation set was used to verify the usefulness of IL-6 as a prognostic predictor for Atezo+Bev. Patient background and tumor factors of the validation group and the training group are presented in Supplementary Table 7. The two parameters were highly correlated (*r* = 0.82), and the equation of covariance showed that the cutoff value of 9.2 used in the Luminex method corresponded to 18.1 pg/mL in the ECLIA method. A comparison of the treatment effect of the validation set divided into two groups by IL-6 18.1 pg/mL is presented in Supplementary Fig. S3b. Although there was no significant difference in the best response, there was a significant difference in both OS and PFS (*p* < 0.001, *p* = 0.029) (Fig.2g–i).

### Comparison of Atezo+Bev and lenvatinib in patients with low serum IL-6 levels

This study demonstrates that IL-6 levels may be a prognostic factor for Atezo+Bev therapy. The distribution of serum IL-6 levels measured using the Luminex assay in the 96 HCC cases is shown in Supplementary Fig. S3a, and a comparison of patient and tumor factors in both groups divided by the IL-6 cutoff value of 9.2 pg/mL is shown in Table 3. When comparing the low IL-6 group with the high IL-6 group, the low IL-6 group had better PS and included cases with good liver reserve, but no significant items were found regarding tumor factors. In addition, among the chemokines verified in this study, IL-8 was significantly lower in the low IL-6 group compared to the high IL-6 group. IL-6 and IL-8 had a weak correlation (Supplementary Fig. 4a). In this background-matched HCC set, no significant difference in best response rate, OS, or PFS was observed between the Atezo+Bev and lenvatinib groups (Supplementary Fig. 5a-c). The IL-6 high group included 11 cases in the Atezo+Bev group and only 4 cases in the lenvatinib group, so it was not possible to compare the therapeutic benefits of Atezo+Bev and lenvatinib in patients with high IL-6 levels. In the IL-6 low group (37 of Atezo+Bev, 44 of lenvatinib), we examined whether lenvatinib or Atezo+Bev treatment was more likely to be beneficial by evaluating prognosis. There were no significant differences in patient demographics or tumor factors (Supplementary Table 8). In evaluating treatment effectiveness, no significant difference was observed in the best response, OS, or PFS between the Atezo+Bev and lenvatinib groups, but in cases with low IL-6 levels, the DCR was higher in the Atezo+Bev group (94.6% vs 86.4%). Although there was no difference in OS between Atezo+Bev and lenvatinib group, PFS was extended in the Atezo+Bev group in that subset (*p* = 0.014) (Supplementary Fig. 5d–f). These results suggest that Atezo+Bev provides therapeutic benefits to patients with low IL-6 levels but not to patients with high IL-6 levels.

**Table 3:** Comparison of baseline characteristics of patients with high- and low-serum IL-6 levels

Parameter		IL-6		
		Low (< 9.2) *n* = 81	High (≥ 9.2) *n* = 15	*p* value
Sex	Male/female	62/19	12/3	0.77
Age (years)		73 (8.43)	75 (11.51)	0.95
Body weight (kg)		61 (12.3)	58 (11.2)	0.82
ECOG performance status	0/1 and 2	75/6	11/4	0.02
Child–Pugh class	A/B	75/6	12/3	0.12
ALBI score		−2.42 (0.42)	−2.19 (0.37)	0.03
Tumor size (mm)		30 (36.5)	30 (37.97)	0.98
Intrahepatic tumor	Single/multiple	6/75	1/14	0.92
Macroscopic vascular invasion		16	3	0.98
Extrahepatic spread		20	7	0.08
BCLC stage	A/B/C	2/49/30	2/6/7	0.09
Etiology of HCC	HBV/HCV/non-viral	9/23/49	2/6/7	0.59
AFP (ng/mL)		24 (75856.6)	26.6 (1782.9)	0.96
DCP (mAU/mL)		333 (120450.5)	1224 (48842.9)	0.07
Angiopoietin-2 (pg/mL)		4336.1 (3016.9)	5662.5 (5677.8)	0.15
CEACAM-1 (pg/mL)		53347.2 (54284.5)	53802.0 (52064.9)	0.73
IL-8 (pg/mL)		37.3 (51.8)	107.0 (56.1)	<0.0001

Data are presented as counts or median (standard deviation)

*AFP* α-fetoprotein; *ALBI score* albumin and bilirubin score; *BCLC* Barcelona Clinic Liver Cancer; *DCP* des-γ-carboxy prothrombin, *HBV* hepatitis B virus; *HCC* hepatocellular carcinoma; *HCV* hepatitis C virus; *IL-6* interleukin-6; *IL-8* interleukin-8

### Analysis of mechanisms

**Fig. 3:**
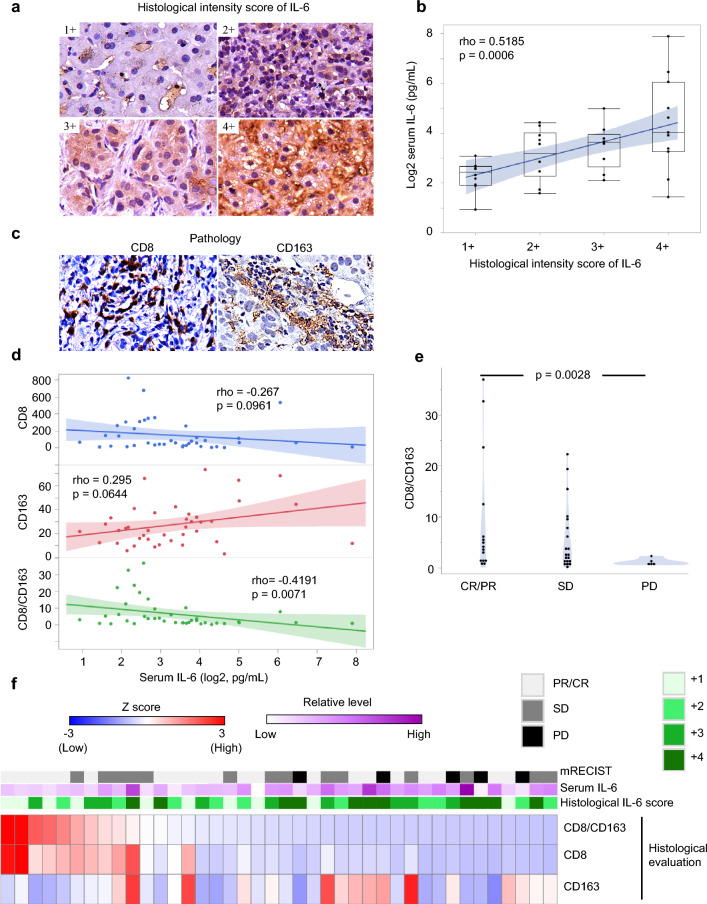
**a** Representative images of immunohistochemistry staining scores (1+, 2+, 3+, 4+) for IL-6 in HCC tumor. **b** Box plots showing the significant positive correlation between immunohistochemistry staining scores of IL-6 and serum IL-6 concentration. **c** Representative image of staining of CD8 (upper left) and CD163 (upper right). **d** A scatter plot showing the correlation between the serum IL-6 concentration and the number of tumor infiltrated CD8- (upper panel, *rho* = −0.267, *p* = 0.0961), CD163- (middle panel, *rho* = 0.295, *p* = 0.0644) positive cells or CD8/CD163-positive cell ratio (lower panel, *rho* = −0.4191, *p* = 0.0071). **e** Violin plots showing a significant difference in tumor-infiltrated CD8- /CD163- positive cell ratio between responder and patients who did not have disease control (*p* = 0.0228). **f** A heatmap summarizing the relationship of the histologically evaluated tumor immune microenvironment, serum IL-6 levels, and clinical response to the Atezo+Bev. Each column represents a single subject and blue–white–red color scale indicates the histologically evaluated tumor-infiltrating CD8 T cell, CD163-positive cells, and CD8/CD163-positive cell ratio. Light gray, gray, and black annotations indicate the best response evaluated by mRECIST PR/CR, SD, and PD, respectively. The white–purple color scale indicates relative serum IL-6 levels, and annotations in tints of green indicate the histological IL-6 scores. *CR*, complete response; *HCC*, hepatocellular carcinoma; *mRECIST*, modified response evaluation criteria in solid tumors; *PD*, progression of disease; *PR*, partial response; *SD*, stable disease

First, the immunostaining of liver biopsy specimens from 40 patients in the Atezo+Bev group revealed a significant positive correlation between serum IL-6 levels and tissue IL-6 expression levels in HCC tumors (Fig. 3a and b). To determine how serum IL-6 affects TME in uHCC, we used CIBERSORT to assess the correlation between immune cell infiltration of tumors and IL-6 expression levels in the TCGA-LIHC cohort and found a positive correlation between tissue IL-6 expression and M2 macrophage counts (Supplementary Fig. 6). From these results, we hypothesized that patients with high serum IL-6 levels are more likely to have a TAM-rich tumor microenvironment. For verification, we evaluated the expression levels of CD163- and CD8-positive cells in the tumor. There was a weak negative correlation between serum IL-6 and tumor-infiltrating CD8-positive cells (*ρ* = −0.267), a weak positive correlation between serum IL-6 and the number of CD163-positive cells (*ρ* = 0.295), and a significant negative correlation between the CD8/CD163-positive cell ratio (*ρ* = −0.42, *p* = 0.00071) (Fig. 3c and d). The CD8/CD163-positive cell ratio is thought to represent the relative activation of TMA. Supporting this idea, the tumor-infiltrating CD8/CD163-positive cell ratio was significantly higher in responding patients than in patients without disease control (*p* = 0.0228) (Fig.3e). Heat maps of 40 cases that underwent IHC are presented in Fig. 3f.

## Discussion

HCC accounts for 90% of primary liver cancers and was the sixth most common cancer and the third leading cause of cancer-related death in 2020 [1]. Although a wide range of treatment options exist for HCC, it is important to select an appropriate systemic therapy to prolong the prognosis of patients with HCC when local therapy fails due to recurrence. This study revealed that patients with high blood IL-6 levels were less likely to benefit from Atezo+Bev therapy. This result is consistent with previous studies, and this study contributes significantly as a validation study [5, 6].

IL-6 plays an important role in the development and progression of HCC [12]. Serum IL-6 levels are higher in patients with HCC compared with healthy controls, and elevated serum IL-6 levels are known to be a risk factor for the development of HCC in patients with chronic liver disease, independent of other risk factors such as hepatitis virus infection [16]. Within tumors, IL-6 is known to stimulate tumor growth, survival, angiogenesis, and evasion of immune detection via IL-6/STAT3 signaling, as well as to enhance and propagate oncogenic signals within the TME, thereby promoting tumorigenesis, invasion, and metastasis [12]. However, IL-6 is also known to correlate with the malignancy potential of the tumor itself, such as in patients with advanced stage HCC or high AFP, and has been reported to contribute to shorter OS regardless of treatment [13, 14, 17].

One of the difficulties in biomarker research is that when a marker is associated with prognosis, it is unclear whether it reflects a response to treatment or a difference in the rate of tumor progression regardless of treatment. In addition, cytokines are known to differ significantly not only by tumor stage but also by etiology [16, 18]. To clarify these points, a comparison was made with a background-matched lenvatinib cohort, excluding confounding factors, which is a unique feature of this paper. Unfortunately, due to the small number of patients with high IL-6 levels in the lenvatinib group, a comparison between lenvatinib and Atezo+Bev in patients with high IL-6 levels was not possible. However, there was no difference in PFS between Atezo+Bev and lenvatinib after PSM, but when limited to patients with low IL-6 levels, PFS for Atezo+Bev was longer. These results may suggest that cases with high IL-6 are not merely more aggressive tumors, but the immune state may also weaken the effect of Atezo+Bev.

In our study, both high serum IL-6 and IL-8 levels were significantly associated with shorter PFS in univariate analysis. However, in multivariate analysis, only high IL-6 was identified as an independent factor. There was a positive correlation between serum IL-6 and IL-8 levels (*p* = 0.0005, *r* = 0.35, Supplementary Fig. 4c), suggesting that IL-6, being the stronger factor, remained as the independent variable. Therefore, we focused on IL-6 as the only factor related to PFS in the subsequent analyses. However, as the scatter plot shows, there were cases where IL-6 was low but IL-8 was high, or vice versa. Moreover, several reports suggest that patients with high blood IL-8 levels may be less likely to benefit from PD-L1 blockade in other cancers [19, 20]. IL-8 is associated with angiogenesis, cell proliferation, invasion, and migration, and is known to correlate with tumor size and grade [17, 21]. It has also been reported that IL-6 and IL-8 are secreted by neutrophils via HSP90α in the TME of HCC and suppress CD8+ T-cell activation [22]. This indicates that combining IL-6 and IL-8 could potentially provide a more accurate prediction of treatment efficacy, and this warrants further investigation in future studies. Numerous studies have revealed that IL-6 induces an immunosuppressive tumor microenvironment. IL-6 exerts its biological effects by binding to the IL-6 receptor (IL-6R). The IL-6R is a complex composed of IL-6Rα (also known as CD126) and glycoprotein 130 (GP130), which is involved in IL-6 signaling. When IL-6Rα binds to IL-6, GP130 homodimerizes, leading to the rapid activation of JAK1, JAK2, and TYK2, which subsequently phosphorylate tyrosine residues within the cytoplasmic domain of GP130. These phosphorylated sites function as docking areas for members of the STAT family of transcription factors (STAT1, STAT3, STAT5). The STAT3-stimulating activity of IL-6 has recently been associated with the suppression of T cell anti-tumor activities. The IL-6-STAT3 signaling pathway counteracts the TCR-dependent enhancement of GZMB, TNF-α, and IFN-γ expression, thereby inhibiting CTL differentiation. Furthermore, IL-6 released in the tumor microenvironment suppresses the differentiation of IFN-γ-producing TH cells by reducing MHC-II surface expression and IL-12 secretion in dendritic cells of tumor-bearing mice. Conversely, the deletion of IL-6 in tumor-bearing mice enhances the anti-tumor activities of effector T cells and inhibits tumor formation in vivo [23]. In addition, IL-6 promotes the recruitment and differentiation of immunosuppressive myeloid cells, such as MDSCs and TAMs [24]. TAMs can weaken the cytotoxic functions of CD8+ T cells and induce their apoptosis [25]. Thus, IL-6 serves as a key player in suppressing CD8+ T cells—either indirectly through the differentiation of suppressive TAMs or directly—ultimately undermining cancer immunity.

Our study showed that in cases with high serum IL-6 levels, IL-6 uptake into the tumor is similarly enhanced within TME, and the CD8/CD163 cell ratio in the tumor is reduced, which may present an immunosuppressive microenvironment. M2 macrophages are representative key players in TAM [26] and act in a tumor-promoting manner through immunosuppressive, angiogenic, and cancer cell infiltration-promoting effects [26]. M2 macrophages are known to be a poor prognostic factor in HCC [27] and have been suggested to be associated with PD-L1 overexpression [28, 29]. Regarding the relationship between M2 macrophages and IL-6, it is known that IL-6 is involved in the polarization of M0 macrophages to M2 macrophages in cancer cells of other organs [30, 31]. Furthermore, in HCC, M2 macrophages have been shown to contribute to tumor progression via the IL-6/STAT3 signaling pathway [32, 33]. Our study indicates that measurement of serum IL-6 may be able to predict the presence of an immunosuppressive TME. To our knowledge, this is the first paper to comprehensively analyze the relationship between serum IL-6 levels and the immune microenvironment and response to treatment in HCC patients treated with Atezo+Bev.

Markers such as CRP, AFP [34, 35], the CRAFITY score derived from CRP and AFP [36], and the neutrophil to lymphocyte ratio [37] have been reported as promising predictors of treatment benefit from immunotherapy in HCC. In our study as well, CRAFITY was identified as a significant marker for progression-free survival (PFS) in univariate analysis. Further investigation of their relevance, including potential combinations with IL-6, is necessary based on findings from larger multi-institutional studies.

Furthermore, early changes in AFP [38–40] and DCP [38, 40] have been reported to be useful in predicting the efficacy of combination immunotherapy. By effectively combining pre-treatment markers with those that assess reactivity after the initiation of treatment, we believe that further improvements in the accuracy of prognosis prediction can be achieved.

In the current study, the IL-6-high group had higher ALBI scores. In the Phase III IMbrave150 study, the response rate to Atezo+Bev tended to decrease with the progression of ALBI grade, with response rates of 32% for ALBI grade 1, 30% for grade 2a, and 25% for grade 2b [41]. Moreover, it has been reported that patients with more advanced ALBI grades prior to treatment have shorter overall survival [42, 43] and PFS [43]. Based on these findings, the poor outcomes observed in cases with high IL-6 levels may not only be due to an immunosuppressive environment but also influenced by reduced hepatic function.

Our analysis of the treatment course after progression (PD) revealed that cases with high IL-6 levels were less likely to reach later-line treatments. Among the cases that started treatment with Atezo+Bev, 10 out of 27 cases with low IL-6 levels were able to reach third-line treatment, while only 2 out of 9 cases with high IL-6 levels were able to do so. During the observation period, among the 70 cases treated with Atezo+Bev as first-line therapy, 28 patients (9 with high IL-6 levels and 19 with low IL-6 levels) died (Supplementary Fig. 7). In the high IL-6 group, the causes of death included 3 patients who died due to the progression of intrahepatic tumors and 3 patients who died from liver failure unrelated to tumor progression (both 10.7%). In contrast, in the low IL-6 group, 9 patients died due to tumor progression (32.1%), and 2 patients died from liver failure unrelated to tumor progression (7.1%), indicating a difference in the causes of death (Supplementary Table 9). These findings suggest that, in addition to the low efficacy of treatment, the decreased reserve capacity may have contributed to the poor prognosis observed in patients with high IL-6 levels.

### Limitations

This study was conducted at a single institution, the number of cases was limited, and sampling bias may exist because it was not possible to examine all tissues in all patients. Furthermore, the cutoff values were evaluated in a limited sample and may change as the number of cases increases. The validity of the cutoff values can be evaluated by increasing the number of cases in the future, but the cutoff values in this study do not deviate significantly compared to previous studies [5, 6]. In this study, there were few cases with high IL-6 levels in the lenvatinib group, and we have not been able to compare the efficacy of Atezo+Bev and lenvatinib in uHCC patients with high IL-6 levels. This is undeniably a limitation when discussing whether Atezo+Bev or lenvatinib is superior in patients with high IL-6 levels. Another limitation of this study is that it was conducted at a single institution. To aim for the generalization of the biomarker, broader validation across different patient populations and institutions is necessary.

## Conclusion

Serum IL-6 levels correlated with the expression of IL-6 in tumors and were thought to be involved in the suppression of tumor immunity in the TME. Measurement of serum IL-6 is useful for predicting the therapeutic effect of Atezo+Bev treatment, which is influenced by the immune environment.

## Supplementary Information

Below is the link to the electronic supplementary material.

Supplementary file1 (DOCX 52 KB)

Supplementary file2 (DOCX 1216 KB)

Supplementary file3 (DOCX 59 KB)

## Abbreviations

AFP: α-Fetoprotein
ALBI score: Albumin and bilirubin score
Atezo+Bev: Atezolizumab plus bevacizumab
BCLC: Barcelona Clinic Liver Cancer
CCL22: CC motif chemokine 22
CEACAM-1: Carcinoembryonic antigen-related cell adhesion molecule 1
CR: Complete response
CRAFITY score: CRP and AFP in immunotherapy score
CT: Computed tomography
CXCL10: C-X-C motif ligand 10
CXCL11: C-X-C motif ligand 11
CXCL5: C-X-C motif ligand 5
CXCL9: C-X-C motif ligand 9
DCP: Des-γ-carboxy prothrombin
DCR: Disease control rate
FDR: False discovery rate
HBV: Hepatitis B virus
HCC: Hepatocellular carcinoma
HCV: Hepatitis C virus
HR: Hazard ratio
ICI: Immune checkpoint inhibitor
IL-10: Interleukin-10
IL-2: Interleukin-2
IL-4: Interleukin-4
IL-6: Interleukin-6
IL-8: Interleukin-8
mRECIST: Modified response evaluation criteria in solid tumors
MRI: Magnetic resonance imaging
MVI: Microvascular invasion
ORR: Overall response rate
OS: Overall survival
PD: Progression of disease
PFS: Progression-free survival
PR: Partial response
ECOG PS: Eastern Cooperative Oncology Group performance status
PSM: Propensity score matching
RDI: Relative dose intensity
ROC: Receiver operating characteristic
SD: Stable disease
SVR: Sustained virological response
TCGA-LIHC: The Cancer Genome Atlas liver hepatocellular carcinoma
TME: Tumor microenvironment
TNF R: Tumor necrosis factor receptor
TNF-alpha: Tumor necrosis factor-alpha
uHCC: Unresectable hepatocellular carcinoma
