# Supplementary material for: Serum IL-6 concentration is a useful biomarker to predict the efficacy of atezolizumab plus bevacizumab in patients with hepatocellular carcinoma
Source: J Gastroenterol. 2024 Dec 9;60(3):328–39. doi: 10.1007/s00535-024-02185-w (PMC11880141; doi:10.1007/s00535-024-02185-w)
Supplement: Supplementary file 1 — Supplementary file1 (DOCX 52 KB) [file 535_2024_2185_MOESM1_ESM.docx]

Supplementary table 1

**Comparison of baseline characteristics of patients in the Atezo+Bev and lenvatinib groups**

|  | Atezo+Bev (n=90) | Lenvatinib (n=58) | p value |
| --- | --- | --- | --- |
| Age , years | 73 [68-82] | 72 [69.5-79.25] | 0.63 |
| Sex |  |  | 0.52 |
| Male | 69 (76.7 %) | 47 (81 %) |  |
| Female | 21 (23.3 %) | 11 (19.0 %) |  |
| Body weight, kg | 62 [54-70] | 59.5 [53.75-70.25] | 0.88 |
| ECOG performance status |  |  | 0.11 |
| 0 | 78 (86.7 %) | 56 (96.6 %) |  |
| 1 | 9 (10.0%) | 2 (3.4 %) |  |
| 2 | 3 (3.3 %) | 0 (0 %) |  |
| Etiology of liver disease |  |  | 0.53 |
| HBV | 11 (12.2 %) | 6 (10.3%) |  |
| HCV | 33 (36.7 %) | 18 (31.0 %) |  |
| HBV＋HCV | 0 (0%) | 1 (56.9 %) |  |
| Non-viral | 46 (51.1 %) | 33 (56.9 %) |  |
| Child-Pugh class |  |  | 0.04 |
| A | 66 (73.3 %) | 2 (3.4 %) |  |
| B | 21 (23.3 %) | 29 (50 %) |  |
| C | 3 (3.3 %) | 27 (46.6 %) |  |
| ALBI score | *-2.33 [-1.88 to -2.66] | *-2.37 [-2.02 to -2.66] | 0.46 |
| mALBI grade |  |  | 0.5 |
| 1 | 25 (27.8 %) | 18 (31.0 %) |  |
| 2a | 26 (28.9 %) | 12 (20.7 %) |  |
| 2b | 33 (36.7 %) | 26 (44.8 %) |  |
| 3 | 6 (6.7 %) | 2 (3.4 %) |  |
| Tumor size, mm | 35 [21.5-60] | 32 [18-70.75] | 0.55 |
| Intrahepatic tumor |  |  |  |
| Single | 13 (14.4 %) | 5 (8.6 %) |  |
| Multiple | 77 (85.6 %) | 53 (91.4 %) |  |
| Macroscopic vascular invasion | 22 (24.4 %) | 14 (24.1 %) | 0.97 |
| Extrahepatic spread | 18 (20.0 %) | 21 (36.2 %) | 0.03 |
| BCLC stage |  |  | 0.38 |
| A | 8 (8.9 %) | 2 (3.4 %) |  |
| B | 46 (51.1 %) | 29 (50.0 %) |  |
| C | 36 (40.0 %) | 27 (46.6 %) |  |
| AFP, ng/ml | 20.85 [3.57-279.8] | 38.05 [4.47-1890.15] | 0.1 |
| DCP, mAU/mL | 542.5 [117.75-4983] | 380 [61-4312.7] | 0.43 |

Note: Data are expressed as number (percentage) or median [interquartile range].

Abbreviations: AFP, α-fetoprotein; ALBI score, albumin and bilirubin score; Atezo+Bev, atezolizumab plus bevacizumab: BCLC, Barcelona-Clinic Liver-Cancer; DCP, des-γ-carboxy prothrombin, HBV, hepatitis B virus; HCC, hepatocellular carcinoma; HCV, hepatitis C virus.Supplementary table 2

**Background of patients who underwent immunohistochemical staining**

|  | n=40 |  |
| --- | --- | --- |
| Age , years | 73 [68.25-83] |  |
| Sex |  |  |
| Male | 31 (77.5 %) |  |
| Female | 9 (22.5 %) |  |
| Body weight, kg | 62 [51.75-65.75] |  |
| ECOG performance status |  |  |
| 0 | 33 (82.5 %) |  |
| 1 | 5 (12.5 %) |  |
| 2 | 2 (5.0 %) |  |
| Etiology of liver disease |  |  |
| HBV | 5 (12.5 %) |  |
| HBV＋HCV | 16 (40.0 %) |  |
| Non-viral | 19 (47.5 %) |  |
| Child-Pugh class |  |  |
| A | 28 (70.0 %) |  |
| B | 10 (25.0 %) |  |
| C | 2 (5.0 %) |  |
| ALBI score | *-2.44 [-1.86 to -2.68] |  |
| mALBI grade |  |  |
| 1 | 14 (35.0 %) |  |
| 2a | 8 (20.0 %) |  |
| 2b | 14 (35.0 %) |  |
| 3 | 4 (10.0 %) |  |
| Tumor size, mm | 45 [20.75-79.25] |  |
| Intrahepatic tumor |  |  |
| Single | 6 (15.0 %) |  |
| Multiple | 34 (85.0 %) |  |
| Macroscopic vascular invasion | 14 (35.0 %) |  |
| Extrahepatic spread | 10 (25.0 %) |  |
| BCLC stage |  |  |
| A | 3 (7.5 %) |  |
| B | 17 (42.5 %) |  |
| C | 20 (50.0 %) |  |
| AFP, ng/ml | 27.1 [3.6-834.2] | |
| DCP, mAU/mL | 752.5 [179.25-8247.25] |  |
| IL-6 ECLIA methods ,pg/mL | 9.05 [5-16] |  |
| IL-6 High/Low (≥18.1/ <18.1 pg/mL) |  |  |
| High | 8 (20.0 %) |  |
| Low | 32 (80.0 %) |  |
| Best response (mRECIST) |  |  |
| CR | 3 (7.5 %) |  |
| PR | 18 (45.0 %) |  |
| SD | 14 (35.0 %) |  |
| PD | 5 (12.5 %) |  |

Note: Data are expressed as number (percentage) or median [interquartile range].

Abbreviations: AFP, α-fetoprotein; ALBI score, albumin and bilirubin score; BCLC, Barcelona-Clinic Liver-Cancer; CR, complete response ; DCP, des-γ-carboxy prothrombin, HBV, hepatitis B virus; HCC, hepatocellular carcinoma; HCV, hepatitis C virus; PD, progression of disease; PR, partial response; SD, stable disease.

**Supplementary table 3.**

**Comparison of baseline characteristics between** **Atezo+Bev training and lenvatinib groups**

|  | Atezo+Bev (n=48) | Lenvatinib (n=48) | p value |
| --- | --- | --- | --- |
| Age , years | 73 [69-81.25] | 73 [70-80.75] | 0.88 |
| Sex |  |  | 0.33 |
| Male | 35 (72.9 %) | 39 (81.3 %) |  |
| Female | 13 (27.1 %) | 9 (18.8 %) |  |
| Body weight, kg | 59.5 [52-66] | 61.5 [54-70.75] | 0.31 |
| ECOG performance status |  |  | 0.09 |
| 0 | 40 (83.3 %) | 46 (95.8 %) |  |
| 1 | 5 (10.4 %) | 2 (4.2 %) |  |
| 2 | 3 (6.3 %) | 0 (0 %) |  |
| Etiology of liver disease |  |  | 0.93 |
| HBV | 6 (12.5 %) | 5 (10.4 %) |  |
| HCV | 14 (29.2 %) | 15 (31.3 %) |  |
| Non-viral | 28 (58.3 %) | 28 (58.3 %) |  |
| Child-Pugh class |  |  | 0.28 |
| A | 45 (93.8 %) | 46 (95.8 %) |  |
| B | 3 (6.3 %) | 2 (4.2 %) |  |
| ALBI score | *-2.36 [-2.61 to -2.08] | *-2.32 [-2.65 to -2.01] | 0.6 |
| mALBI grade |  |  | 0.17 |
| 1 | 13 (27.1 %) | 14 (29.2 %) |  |
| 2a | 18 (37.5 %) | 10 (20.8 %) |  |
| 2b | 17 (35.4 %) | 22 (45.8 %) |  |
| 3 | 0 (0 %) | 2 (4.2 %) |  |
| Tumor size, mm | 29 [18.25-55] | 34.5 [20.25-71] | 0.57 |
| Intrahepatic tumor |  |  | 0.69 |
| Single | 3 (6.3 %) | 4 (8.3 %) |  |
| Multiple | 45 (93.8 %) | 44 (91.7 %) |  |
| Macroscopic vascular invasion | 9 (18.8 %) | 10 (20.8 %) | 0.8 |
| Extrahepatic spread | 12 (25.9 %) | 15 (31.3 %) | 0.5 |
| BCLC stage |  |  | 0.97 |
| A | 2 (4.2 %) | 2 (4.2 %) |  |
| B | 28 (58.3 %) | 27 (56.3 %) |  |
| C | 18 (37.5 %) | 19 (39.6 %) |  |
| AFP, ng/ml | 14.45 [3-406.15] | 38.0 [5.3-1778.25] | 0.07 |
| DCP, mAU/mL | 651.5 [111.5-2655.5] | 380 [59-3454.25] | 0.58 |

Note: Data are expressed as number (percentage) or median [interquartile range].

Abbreviations: AFP, α-fetoprotein; ALBI score, albumin and bilirubin score; BCLC, Barcelona-Clinic Liver-Cancer; DCP, des-γ-carboxy prothrombin; HBV, hepatitis B virus; HCC, hepatocellular carcinoma; HCV, hepatitis C virus.

**Supplementary table 4**

**Comparison of baseline characteristics of patients in the HCC and Control groups**

|  | Control (n=20) | HCC (n=96) | p value |
| --- | --- | --- | --- |
| Age , years | 74.5 [69-78.75] | 73 [69-80.75] | 0.87 |
| Sex |  |  | 0.25 |
| Male | 13 (65.0 %) | 74 (77.1 %) |  |
| Female | 7 (35.0 %) | 22 (22.9 %) |  |
| Body weight, kg | 66.5 [56.85-74.65] | 60.5 [53.25-68.75] | 0.06 |
| Etiology of liver disease |  |  | 0.91 |
| HBV | 2 (10.0 %) | 11 (11.5 %) |  |
| HCV | 7 (35.0 %) | 29 (30.2 %) |  |
| Non-viral | 11 (55.0 %) | 56 (58.3 %) |  |
| Child-Pugh class |  |  | 0.17 |
| A | 16 (80.0 %) | 87 (90.6 %) |  |
| B | 4 ( 20.0 %) | 9 (9.4 %) |  |
| ALBI score | *-2.39 [-1.94 to-2.59] | *-2.36 [-2.03 to -2.64] | 0.73 |
| mALBI grade |  |  | 0.43 |
| 1 | 4 (20.0 %) | 27 (28.1 %) |  |
| 2a | 8 (40.9 %) | 28 (29.2 %) |  |
| 2b | 6 (30.0 %) | 39 (40.6 %) |  |
| 3 | 1 (5.0 %) | 2 (2.1 %) |  |

Note: Data are expressed as number (percentage) or median [interquartile range].

Abbreviations: ALBI score, albumin and bilirubin score; HBV, hepatitis B virus; HCV, hepatitis C virus.

Supplementary table 5

**Comparison of chemokines between control and PSM-HCC groups**

|  | Control group (n=20) |  | HCC group (n=96) | p value |
| --- | --- | --- | --- | --- |
| Angiopoietin-2 | 3272 [29724.6-46018.5] |  | 4393 [3339.8-6551.1] | 0.007 |
| CCL22 | 482 [300.8-699.4] |  | 455.4 [280.9-549.2] | 0.67 |
| CEACAM-1 | 39776 [29724.6-46018.5] |  | 52835.7 [40448.1-85700.2] | 0.001 |
| CXCL5 | 636.5 [388.9-891.4] |  | 633.2 [395.3-831.6] | 0.81 |
| CXCL10 | 69.1 [47.3-87.3] |  | 68.7 [49.9-107.7] | 0.49 |
| CXCL11 | 23.3 [10.0-37.9] |  | 30.4 [15.9-44.9] | 0.21 |
| Galectin-9 | 6887.9 [5563.3-10770.4] |  | 7986.6 [6355.8-9814.8] | 0.41 |
| IL-6 | 2.4 [2.0-4.7] |  | 4.4 [2.0-7.8] | 0.026 |
| IL-8 | 27.4 [16.8-44.6] |  | 43.2 [28.0-91.6] | 0.003 |
| IL-10 | 20.6 [14.7-31.0] |  | 25.8 [15.2-40.2] | 0.46 |
| TNF-alpha | 8.9 [6.3-15.7] |  | 10.5 [7.3-18.4] | 0.34 |
| TNF RI | 1761.6 [1361.3-2476.6] |  | 2128.7 [1665.3-2590.7] | 0.12 |

Note: Data are expressed as number (percentage) or median [interquartile range].

Abbreviations: CCL22, CC motif chemokine 22; CEACAM-1, carcinoembryonic antigen related cell adhesion molecule 1; CXCL10, C-X-C motif ligand 10; CXCL11, C-X-C motif ligand 11; CXCL5, C-X-C motif ligand 5; IL-10, interleukin-10; IL-6, interleukin-6; IL-8, interleukin-8; TNF RⅠ; tumor necrosis factor receptor Ⅰ; TNF-alpha, tumor necrosis factor-alpha.

Supplementary table 6

**Logistic regression analysis of factors related to PD in lenvatinib group**

|  | Lenvatinib | | | | |
| --- | --- | --- | --- | --- | --- |
|  | Cut off | Odds rate | 95% CI | p value | q value |
| Angiopoietin-2 | ≥4573.5, 4573.5 | 0.66 | (0.11-4.05) | 0.66 | 0.88 |
| CEACAM-1 | ≥48768.5, 48768.5 | 0.45 | (0.08-2.53) | 0.35 | 0.7 |
| IL-6 | ≥2.0, 2.0 | 0.25 | (0.02-3.36) | 0.26 | 1.04 |
| IL-8 | ≥40.9, 40.9 | 1.1 | (0.2-6.09) | 0.91 | 0.91 |

Abbreviations: CEACAM-1, carcinoembryonic antigen related cell adhesion molecule 1; IL-6, interleukin-6; IL-8, interleukin-8.

Supplementary table 7

**Baseline characteristics of patients in Atezo+Bev PSM-HCC and validation group**

|  | Atezo+Bev | |
| --- | --- | --- |
|  | PSM-HCC (n=48) | Validation (n=42) |
| Age, years | 73 [69-81.25] | 74 [66-82] |
| Sex |  |  |
| Male | 35 (72.9 %) | 34 (81.0 %) |
| Female | 13 (27.1 %) | 8 (19.0 %) |
| Body weight, kg | 59.5 [52-66] | 61.5 [56-73] |
| ECOG performance status |  |  |
| 0 | 40 (83.3 %) | 38 (90.5 %) |
| 1 | 5 (10.4 %) | 4 (9.5 %) |
| 2 | 3 (6.3 %) | 0 (0 %) |
| Etiology of liver disease |  |  |
| HBV | 6 (12.5 %) | 5 (11.9 %) |
| HCV | 14 (29.2 %) | 19 (45.2 %) |
| Non-viral | 28 (58.3 %) | 18 (42.9 %) |
| Child-Pugh class |  |  |
| A | 45 (93.8 %) | 21 (50.0 %) |
| B | 3 (6.3 %) | 18 (42.9 %) |
| ALBI score | *-2.36 [-2.61 to -2.08] | *-2.03 [-2.68 to -1.67] |
| mALBI grade |  |  |
| 1 | 13 (27.1 %) | 12 (28.6 %) |
| 2a | 18 (37.5 %) | 8 (19.0 %) |
| 2b | 17 (35.4 %) | 16 (38.1 %) |
| 3 | 0 (0 %) | 6 (14.3 %) |
| Tumor size, mm | 29 [18.25-55] | 50 [30-60] |
| Intrahepatic tumor |  |  |
| Single | 3 (6.3 %) | 10 (23.8 %) |
| Multiple | 45 (93.8 %) | 32 (76.2 %) |
| Macroscopic vascular invasion | 9 (18.8 %) | 13 (31.0 %) |
| Extrahepatic spread | 12 (25.0 %) | 6 (14.3 %) |
| BCLC stage |  |  |
| A | 2 (4.2 %) | 6 (14.3 %) |
| B | 28 (58.3 %) | 18 (42.9 %) |
| C | 18 (37.5 %) | 18 (42.9 %) |
| AFP, ng/ml | 14.45 [3.0-406.15] | 34.25 [3.575-198.8] |
| DCP, mAU/mL | 651.5 [111.5-2655.5] | 364 [117.75-6952] |

.Note: Data are expressed as number (percentage) or median [interquartile range] .

Abbreviations: AFP, α-fetoprotein; ALBI score, albumin and bilirubin score; BCLC, Barcelona-Clinic Liver-Cancer; DCP, des-γ-carboxy prothrombin; HBV, hepatitis B virus; HCC, hepatocellular carcinoma; HCV, hepatitis C virus.

**Supplementary table 8. Comparison of patient demographics of Atezo+Bev and Lenvatinib group with low serum IL-6 levels**

|  | Atezo+Bev (n=37) | Lenvatinib (n=44) | p value |
| --- | --- | --- | --- |
| Age , years | 73 [68-79] | 73.5 [70.25-80.75] | 0.55 |
| Sex |  |  | 0.49 |
| Male | 27 (73.0 %) | 35 (79.5 %) |  |
| Female | 10 (27.0 %) | 9 (20.5 %) |  |
| Body weight, kg | 60 [52-66.5] | 61.5 [54-69.75] | 0.51 |
| ECOG performance status |  |  | 0.11 |
| 0 | 32 (86.5 %) | 43 (97.7 %) |  |
| 1 | 4 (10.8 %) | 1 (2.3 %) |  |
| 2 | 1 (2.7 %) | 0 (0 %) |  |
| Etiology of liver disease |  |  | 0.67 |
| HBV | 5 (13.5 %) | 4 (9.1 %) |  |
| HCV | 9 (24.3 %) | 14 (31.8 %) |  |
| Non-viral | 23 (62.2 %) | 26 (59.1 %) |  |
| Child-Pugh class |  |  | 0.52 |
| A | 35 (94.6 %) | 40 (90.9 %) |  |
| B | 2 (5.4 %) | 4 (9.1 %) |  |
| ALBI score | *-2.45 [-2.70 to -2.10] | *-2.41 [-2.78 to -2.02] | 0.61 |
| Tumor size, mm | 28 [17-55] | 34.5 [21-71] | 0.38 |
| Intrahepatic tumor |  |  | 0.82 |
| Single | 3 (8.1 %) | 3 (6.8 %) |  |
| Multiple | 34 (91.9 %) | 41 (93.2 %) |  |
| Macroscopic vascular invasion | 8 21.6 %) | 8 (18.2 %) |  |
| Extrahepatic spread | 8 21.6 %) | 12 (27.3 %) |  |
| BCLC stage |  |  | 0.98 |
| A | 1 (2.7 %) | 1 (2.3 %) |  |
| B | 22 (59.5 %) | 27 (61.4 %) |  |
| C | 14 (37.8 %) | 27 (61.4 %) |  |
| AFP, ng/ml | 7.4 [3.2-378.3] | 38.0 [5.3-1778.25] | 0.07 |
| DCP, mAU/mL | 333 [84.5-1521.5] | 380 [59-3454.25] | 0.91 |

Note: Data are expressed as number (percentage) or median [interquartile range].

Abbreviations: AFP, α-fetoprotein; ALBI score, albumin and bilirubin score; BCLC, Barcelona-Clinic Liver-Cancer; DCP, des-γ-carboxy prothrombin, HBV, hepatitis B virus; HCC, hepatocellular carcinoma; HCV, hepatitis C virus.

**Supplementary table 9. Causes of death according to serum IL-6 levels and tratment**

|  | Atezo+Bev(n=28) | | |  | | | Lenvatinib (n=34) | | | |
| --- | --- | --- | --- | --- | --- | --- | --- | --- | --- | --- |
| Cause of death | IL-6 High (n=9) | IL-6 Low (n=19) |  | | | IL-6 High (n=3) | | | IL-6 Low (n=31) |  |
| Hepatocellular carcinoma related deaths |  |  |  | |  | | |  | |  |
| Hepatocellular carcinoma progression or liver failure | 3 (10.7 %) | 9 (32.1 %) |  | | 2 (5.9 %) | | | 25 (73.5 %) | |  |
| Liver failure due to decreased liver function with continued treatment | 3 (10.7 %) | 2 (7.1 %) |  | | 0 (0 %) | | | 2 (5.9 %) | |  |
| Respiratory failure due to exacerbation of lung metastases | 0 (0 %) | 1 (3.6 %) |  | | 0 (0 %) | | | 1 (2.9 %) | |  |
| Respiratory failure due to exacerbation of interstitial pneumonia with immune-related adverse events | 0 (0 %) | 0 (0 %) |  | | 0 (0 %) | | | 1 (2.9 %) | |  |
| Right heart failure due to right atrial tumor plug | 0 (0 %) | 1 (3.6 %) |  | | 0 (0 %) | | | 0 (0 %) | |  |
| Non- Hepatocellular carcinoma related deaths |  |  |  | |  | | |  | |  |
| Gastrointestinal bleeding | 1 (3.6 %) | 3 (10.7 %) |  | | 0 (0 %) | | | 0 (0 %) | |  |
| Infection | 0 (0 %) | 1 (3.6 %) |  | | 1 (2.9 %) | | | 2 (5.9 %) | |  |
| Spontaneous bacterial peritonitis | 0 (0 %) | 1 (3.6 %) |  | | 0 (0 %) | | | 1 (2.9 %) | |  |
| Necrotizing fasciitis | 0 (0 %) | 0 (0 %) |  | | 1 (2.9 %) | | | 0 (0 %) | |  |
| Aspiration pneumonia | 0 (0 %) | 0 (0 %) |  | | 0 (0 %) | | | 1 (2.9 %) | |  |
| Renal failure due to progressive congestive heart failure | 0 (0 %) | 1 (3.6 %) |  | | 0 (0 %) | | | 0 (0 %) | |  |
| Unknown | 2 (7.1 %) | 1 (3.6 %) |  | | 0 (0 %) | | | 0 (0 %) | |  |

Note: Data are expressed as number (percentage).
