# Supplementary material for: Serum IL-6 concentration is a useful biomarker to predict the efficacy of atezolizumab plus bevacizumab in patients with hepatocellular carcinoma
Source: J Gastroenterol. 2024 Dec 9;60(3):328–39. doi: 10.1007/s00535-024-02185-w (PMC11880141; doi:10.1007/s00535-024-02185-w)
Supplement: Supplementary file 2 — Supplementary file2 (DOCX 1216 KB) [file 535_2024_2185_MOESM2_ESM.docx]

a


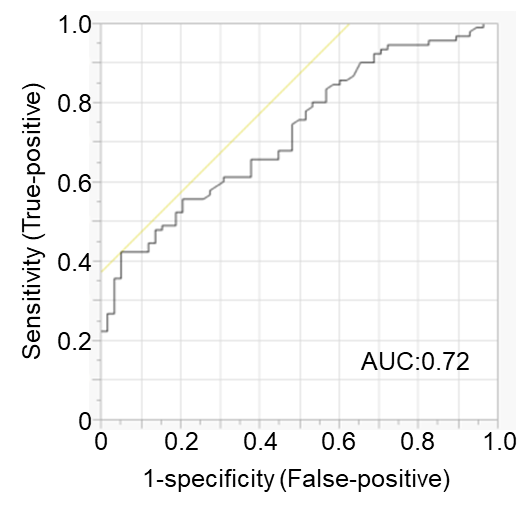


b


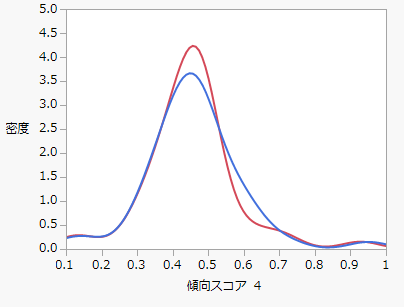

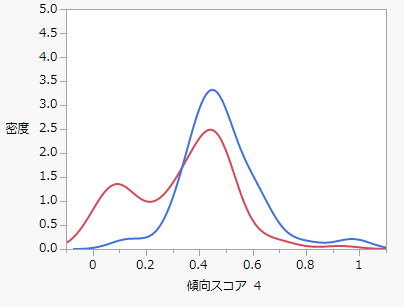


Unadjusted sample

Adjusted sample

Distance

Distance

Density

5.0

4.0

3.0

2.0

1.0

0

1.0

0.8

0.6

0.4

0.2

1.0

0.8

0.6

0.4

0.2

0

Density

5.0

4.0

3.0

2.0

1.0

0

c


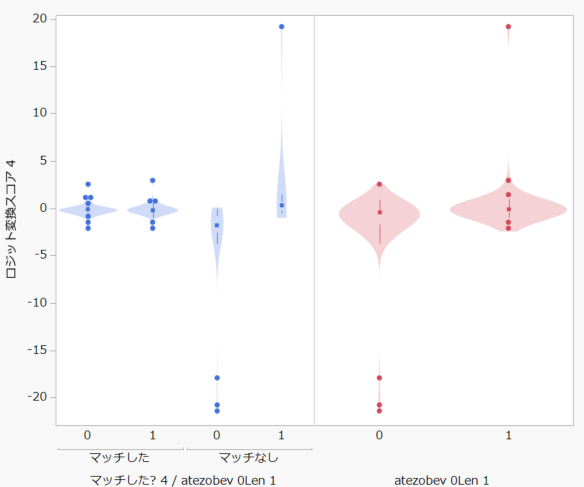


Unadjusted sample

Adjusted sample

Logit scale of propensity score

Atezo/Bev

Atezo+Bev

Lenvatinib

Atezo+Bev

Lenvatinib

Lenvatinib

20

10

-10

-20

0

Matched

Unmatched

**Supplementary figure S1.** Area under the curve calculated for propensity score matching, distributional balance for distance, and violin plots of matched groups. (a) The area under the curve calculated from the receiver operating characteristics curve was 0.72423 (p<0.0071). (b) The distribution balance for the model before and after PSM illustrated a good match between groups after PSM. (c) Violin plots of matched groups. Violin plots of the matched and unmatched groups as well as the groups before matching showed the distribution of dots based on the logit scale of the propensity score, which can be compared between matched groups.

a


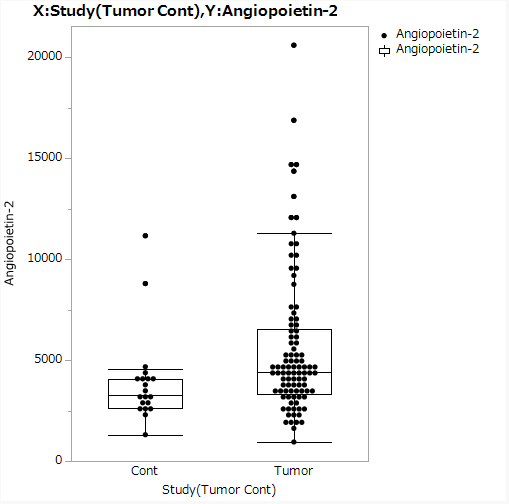


Control group

15000

10000

5000

0

Angiopoietin-2

p = 0.007

20000

HCC group

b


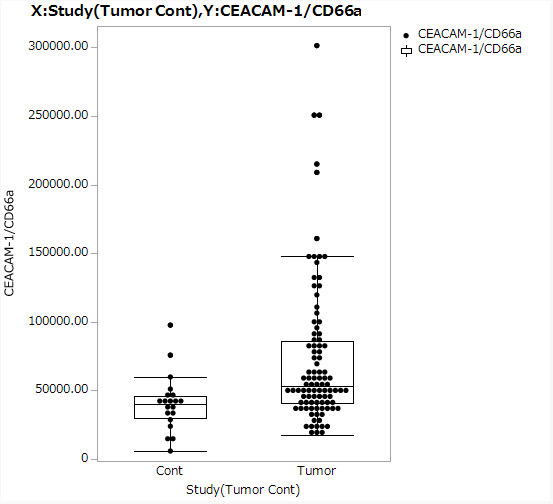


300000

250000

150000

100000

0

200000

50000

CEACAM-1

p = 0.001

HCC group

Control group

d

c


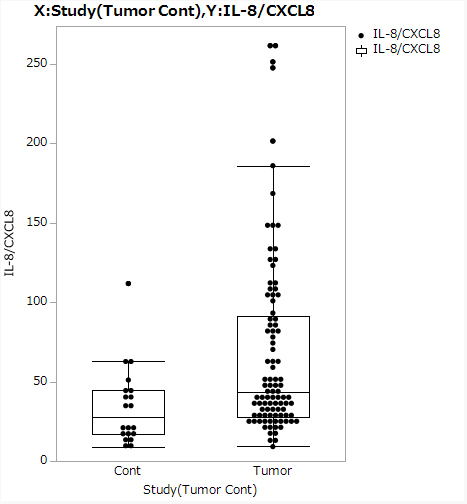


250

200

150

100

50

0

IL-8

p = 0.003


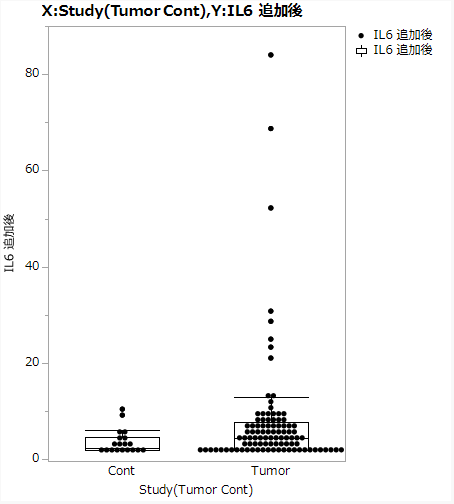


80

60

40

20

0

IL-6

p =0.026

HCC group

Control group

HCC group

Control group

**Supplementary figure S2.** Comparison of Angiopoietin-2, CEACAM-1, IL-6, and IL-8 between control and HCC groups. The HCC group included 96 patients in the Atezo+Bev training group and the lenvatinib group included, and the control group included 20 patients. Among the chemokines measured in this study, Angiopoietin-2, CEACAM-1, IL-6, and IL-8 were significantly higher in the HCC group than in the control group. (a) Scatter plot of serum Angiopoietin-2 in control and HCC group (p=0.007). (b) Scatter plot of serum CEACAM-1 in control and HCC group (p=0.001). (c) Scatter plot of serum IL-6 in control and HCC group (p=0.026). (d) Scatter plot of serum IL-8 in control and HCC group (p=0.003).

a

b


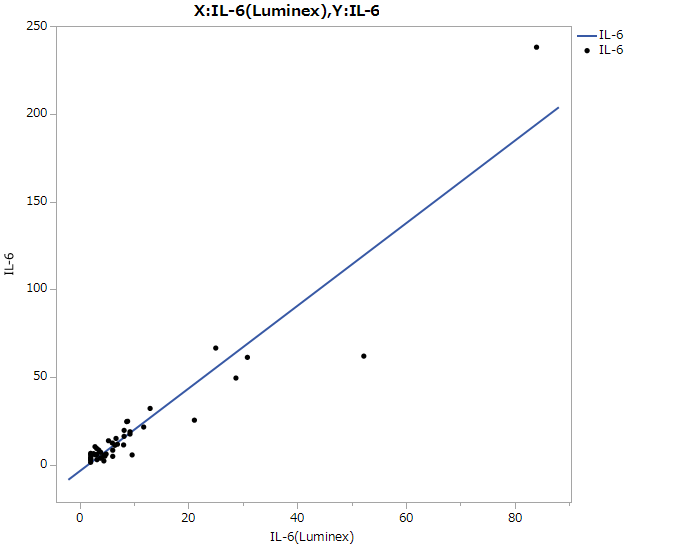


r = 0.82

Y = -3.47 + X×2.35

Cutoff=18.1

Cutoff=9.2

IL-6 (Luminex)

IL-6 (ECLIA)

250

200

150

100

0

50

80

60

40

20

0

p < 0.001


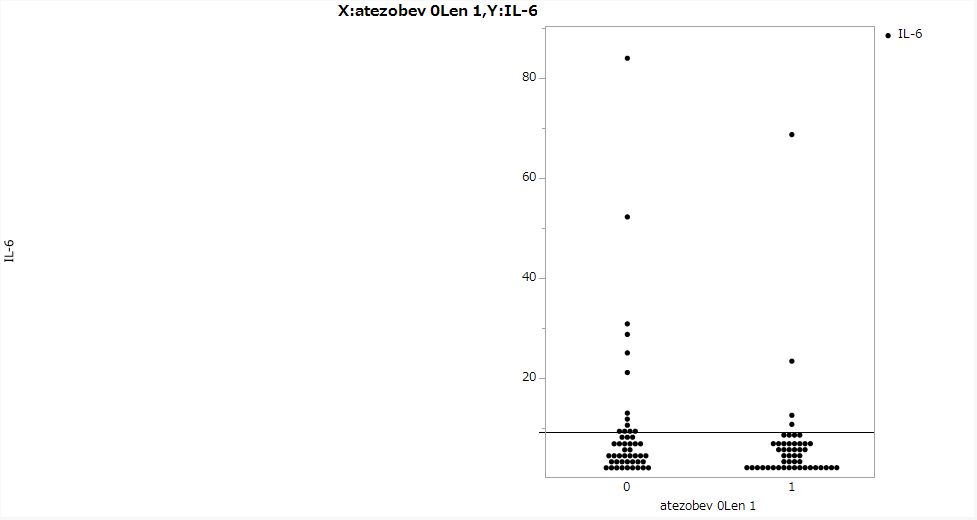


80

60

40

20

0

Atezo+Bev

Lenvatinib

9.2

**Supplementary figure 3**. Scatter plot of serum IL-6. (a) Scatter plot of serum IL-6 in the Atezo + Bev training group and lenvatinib group. (b) Scatter plot of serum IL-6 measured by Luminex and ECLIA methods (p < 0.001, r = 0.82). The cutoff value for IL-6 in the ECLIA method, which corresponds to the Luminex method, was 18.1 pg/mL.

a


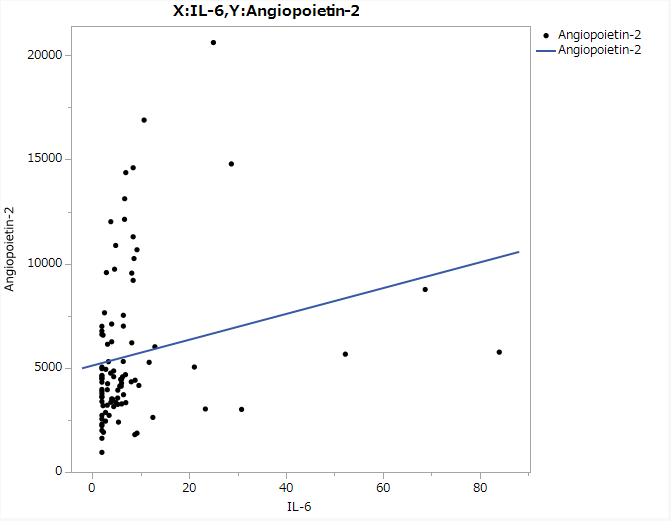


80

60

40

20

0

IL-6 (pg/mL)

Angiopoietin-2 (pg/mL)

5000

20000

10000

0

15000

r = 0.21

p = 0.038

b


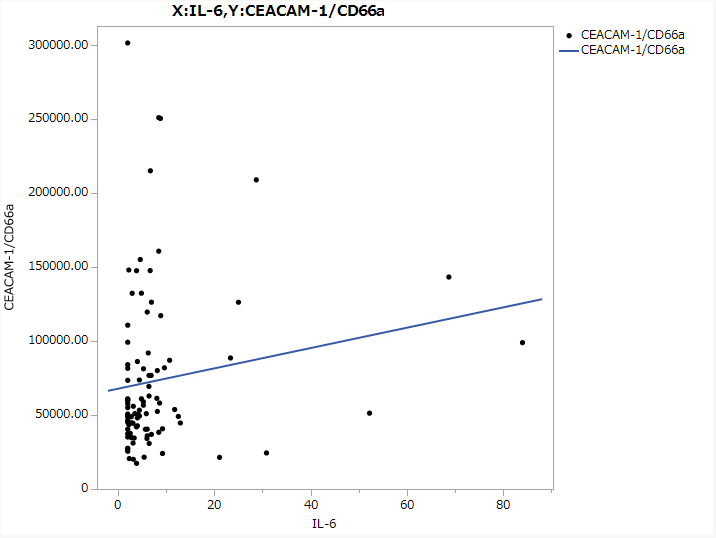


80

60

40

20

0

IL-6 (pg/mL)

CEACAM-1 (pg/mL)

100000

300000

200000

0

r = 0.16

p = 0.12

c


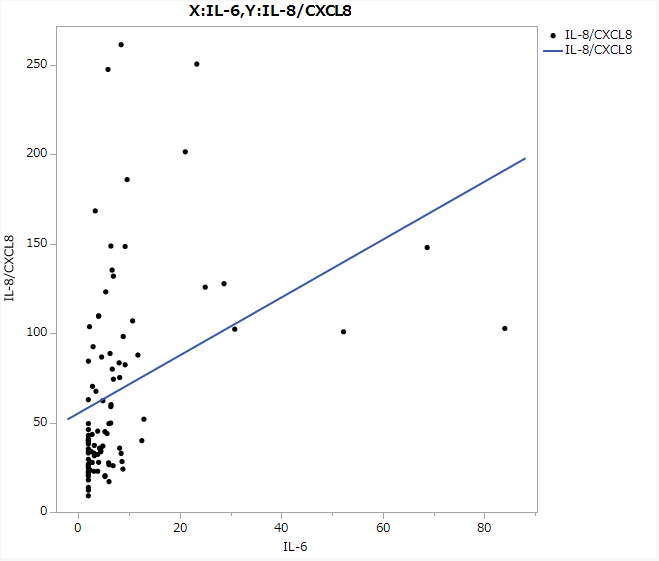


r = 0.35

p = 0.0005

250

200

150

100

0

50

80

60

40

20

0

IL-6 (pg/mL)

IL-8 (pg/mL)

d


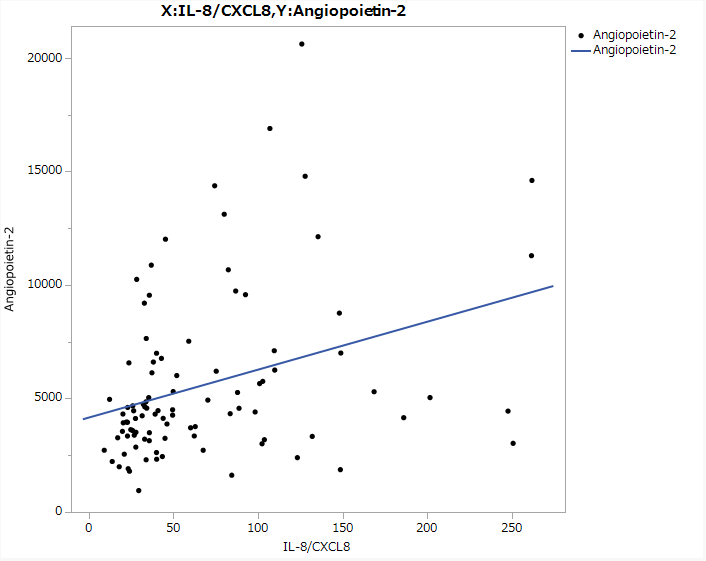


Angiopoietin-2 (pg/mL)

5000

20000

10000

0

15000

250

200

150

100

0

50

IL-8 (pg/mL)

r = 0.34

p = 0.0008

e


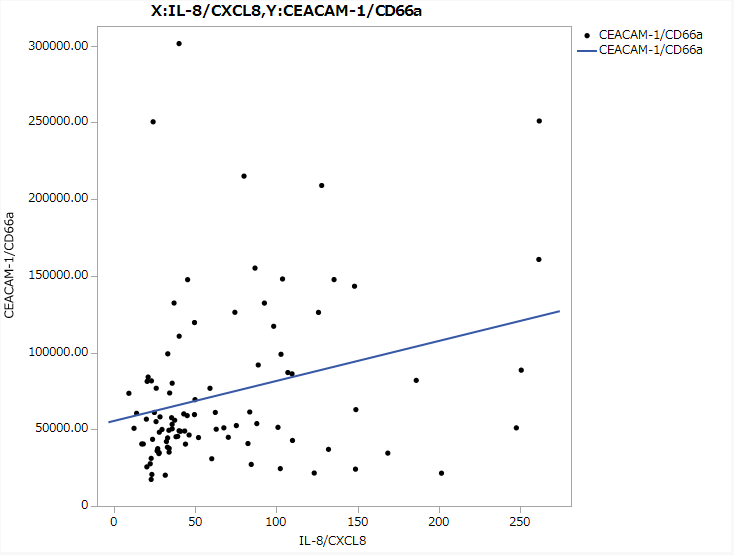


250

200

150

100

0

50

IL-8 (pg/mL)

CEACAM-1 (pg/mL)

100000

300000

200000

0

r = 0.28

p = 0.0058

f


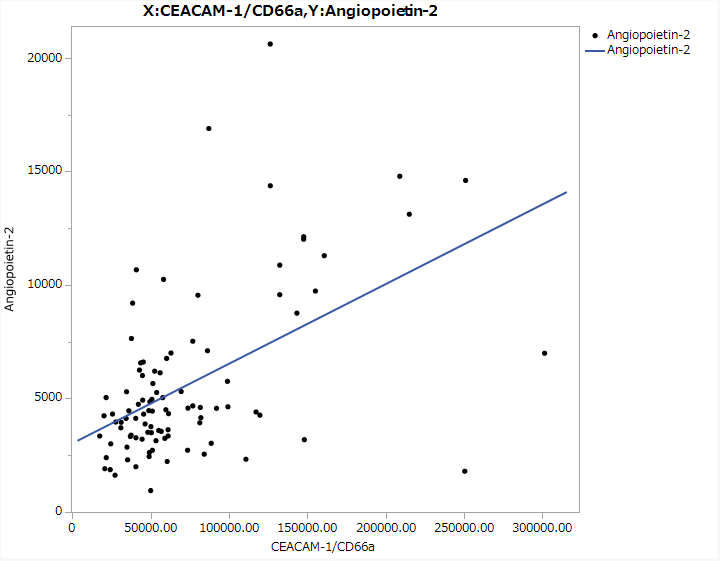


Angiopoietin-2 (pg/mL)

5000

20000

10000

0

15000

CEACAM-1 (pg/mL)

0

100000

200000

300000

r = 0.52

p < 0.0001

**Supplementary figure 4.** Correlation between Angiopoietin-2, CEACAM-1, IL-6, and IL-8. (a) Scatter plot of serum Angiopoietin-2 and IL-6 (p = 0.0381, r = 0.21). (b) Scatter plot of serum CEACAM-1 and IL-6 (p = 0.12, r = 0.16). (c) Scatter plot of serum IL-8 and IL-6 (p = 0.0005, r = 0.35). (d) Scatter plot of serum Angiopoietin-2 and IL-8 (p = 0.0008, r = 0.34). (e) Scatter plot of serum CEACAM-1 and IL-8 (p = 0.0058, r = 0.28). (f) Scatter plot of serum Angiopoietin-2 and CEACAM-1 (p <0.0001, r = 0.52).

**
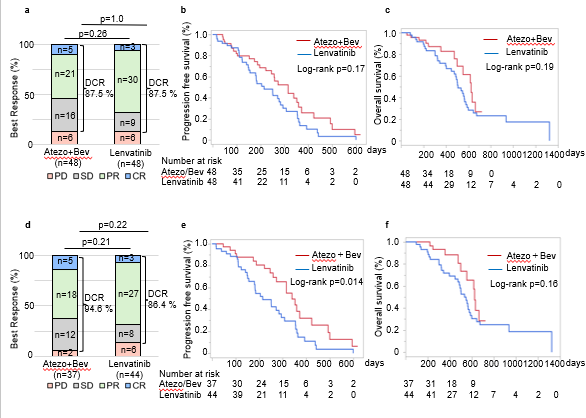
**

**Supplementary figure 5. Comparison of therapeutic responses to high and low serum IL-6 levels in Atezo+Bev and lenvatinib group.**

Best response to Atezo+Bev and lenvatinib after propensity score matching (a) and Kaplan-Meier curves for progression-free survival (PFS) (b) and overall survival (OS) (c). (d-f) The analysis in the low serum IL-6 subgroup. ​​Best response to Atezo+Bev and lenvatinib (d) and Kaplan-Meier curves for progression-free survival (PFS) (e) and overall survival (OS) (f).

-log_10_*P* value

Spearman’s rho


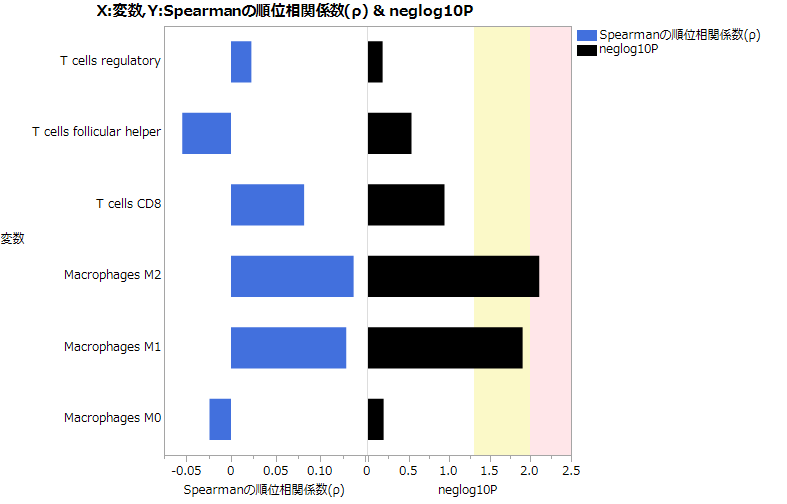


1.5

0.5

1.0

2.0

0

0.10

0

-0.05

0.05

T cells

regulatory

T cells follicular

helper

T cells CD8

Macrophages

M2

Macrophages

M1

Macrophages

M0

**Supplementary figure 6.** Correlation between IL-6 expression and immune cell population. Blue and black bars indicate Spearman’s ρ and -log10P values, respectively. Bars extending to the yellow and red areas indicate p values between 0.01 and 0.05 and <0.01, respectively.


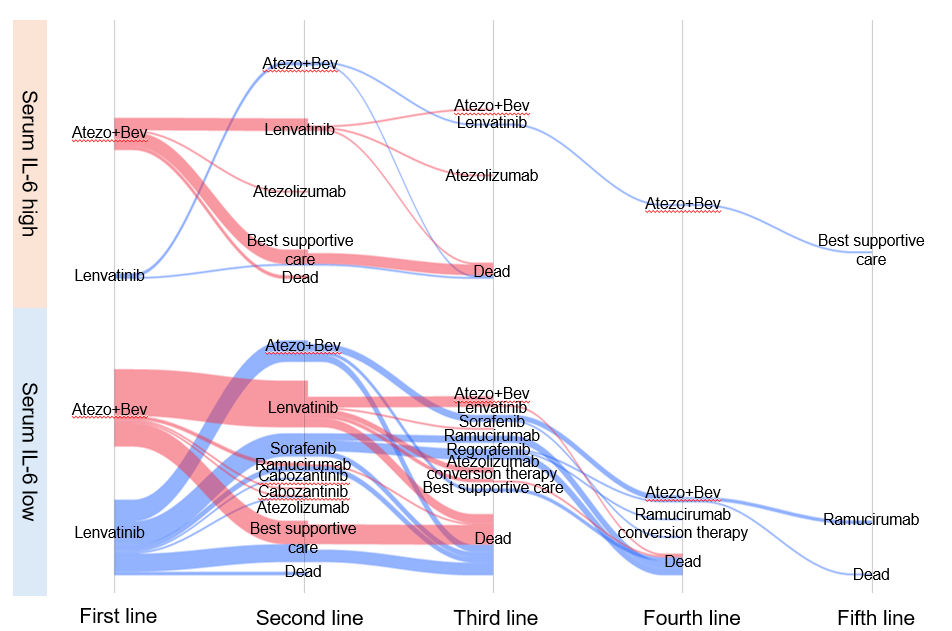


**Supplementary figure 7.** Transition of treatment methods after progression disease depending on serum IL-6 levels. Conversion therapy include local therapy (RFA/MWA, SBRT, surgery).
