# Supplementary material for: Serum IL-6 concentration is a useful biomarker to predict the efficacy of atezolizumab plus bevacizumab in patients with hepatocellular carcinoma
Source: J Gastroenterol. 2024 Dec 9;60(3):328–39. doi: 10.1007/s00535-024-02185-w (PMC11880141; doi:10.1007/s00535-024-02185-w)
Supplement: Supplementary file 3 — Supplementary file3 (DOCX 59 KB) [file 535_2024_2185_MOESM3_ESM.docx]

**Supplementary Information**

**Article title**

Serum IL-6 concentration is a useful biomarker to predict the efficacy of Atezo+Bev in patients with hepatocellular carcinoma

**Journal name**

Journal of Gastroenterology

**Author names and affiliations**

Ryoichi Miura^1^, Atsushi Ono^1^, Kenji Yamaoka^1^, Yasutoshi Fujii^2^, Shinsuke Uchikawa^1^, Hatsue Fujino^1^, Eisuke Murakami^1^, Tomokazu Kawaoka^1^, Daiki Miki^1^, Masataka Tsuge^1^, Takeshi Kishi^3^, Waka Ohishi^4^, Clair. Nelson Hayes^1^, Naoya Sakamoto^5^, Shiro Oka^1^

1 Department of Gastroenterology, Hiroshima University Hospital, Hiroshima, Japan.

2 Department of Clinical Oncology, Graduate School of Biomedical and Health Sciences, Hiroshima University, Hiroshima, Japan.

3 Biosample Research Center, Radiation Effects Research Foundation, Hiroshima, Japan.

4 Department of Clinical Studies, Radiation Effects Research Foundation, Hiroshima, Japan.

5 Department of Pathology and Clinical Laboratories, National Cancer Center-Hospital East, Kashiwa, Chiba, Japan.

**Corresponding author**

Atsushi Ono, MD, PhD

Department of Gastroenterology, Graduate School of Biomedical & Health Sciences, Hiroshima University

Postal address: Hiroshima 734-8551, Japan

Phone: +81-82-257-5191

Fax number: +81-82-257-5194

Email: [atsushi-o@hiroshima-u.ac.jp](mailto:atsushi-o@hiroshima-u.ac.jp)

Supplemental methods

***Patients after propensity matching score***

The propensity score was calculated using a logistic regression model. Covariates entered into the propensity model included etiology of HCC, Child-Pugh class, maximum tumor size, tumor number, macrovascular and microvascular invasion (MVI), satellite nodules, and Barcelona-Clinic Liver-Cancer (BCLC) stage. PSM was performed using a 1:1 matching method with a caliper width of 0.2, and the area under the curve calculated from the receiver operating characteristics curve was 0.72 (p<0.0071) Supplementary Figure S1. A total of 96 patients (48 each from the Atezo + Bev and lenvatinib groups) matched for patient background and tumor factors using propensity scores were selected and defined as the propensity score matched PSM-HCC group.

***Sample Collection***

Venous blood samples were collected prior to the first dose of Atezo+Bev and lenvatinib. Samples were separated into serum after centrifugation and stored at -80°C. Non-HCC samples were collected at appropriate times from HCV patients who achieved sustained virological response (SVR) and HBV cases in which hepatitis had subsided by administration of nucleic acid analogs or other regimens.

***Immunohistochemistry (IHC)***

The primary antibodies used were as follows: CD163 1:500 abcam EPR19518, CD8 1:500 abcam ab217344, IL-6 1:500 abcam ab9324. The secondary antibodies were as follows: SignalStain Boost IHC Detection Reagent #8114, #8125 Cell Signaling Technology, Danvers, MA, USA, and the DAB working solution was SignalStain DAB Substrate Kit #8059 (Cell Signaling Technology, Danvers, MA, USA). IHC staining was performed as previously described [1]. Positive cell counts were assessed using the color deconvolution function of ImageJ version 1.54i 03 (https://imagej.net/ij/). The number of CD8 positive cells in the tumor was assessed at 100x magnification, and the number of CD163 positive cells was calculated by selecting up to three areas of strong intra-tumoral infiltration at 200x magnification and measuring the average. The staining intensity of immunohistochemical staining for IL-6 was quantified using ImageJ software and classified as 1+, 2+, or 3+4+, with the first, second, and third quartiles as cutoffs. Histological evaluation was performed independently by two pathologists and two hepatologists who were blinded to the clinical course of the patients.

***Assessment of profiles of circulating cytokines/chemokines***

Multi-analyte profiling of baseline serum cytokines was performed using the Human Luminex® assay kit (R&D systems, Minneapolis, MN, USA) according to the manufacturer's instructions. The panel design used the Luminex assay customization tool (https://www.rndsystems.com/luminex/analytes) to select from among cytokines that have been reported to be associated with clinical outcomes such as response or tolerance to immunotherapy at the same dilution. A total of 17 serum cytokines (Angiopoietin-2[2], CCL22 [3], CEACAM-1 [4], CXCL5 [4] [5], CXCL9 [6, 7], CXCL10 [6] [8], CXCL11 [6], Galactin-9 [9], Granzyme B[7], IFN-gamma[10], IL-2[10], IL-4 [11], IL-6 [10-12], IL-8[12], IL-10 [11, 12], TNF-alpha [10, 11], TNF RI[10]) were selected. Assays were evaluated using a Luminex 200 analyzer based on Luminex xMAP technology, performed at the Radiation Effects Research Foundation, and blinded with respect to patient characteristics and study endpoints, as in previous reports [13]. The values below the lower limit of the standard curve were treated as values at the lower limit of the standard. The results were registered in the GEO database (GSE261672). Cytokines that entered the measurable range in less than 50% of all cases were excluded from the analysis : CXCL9, Granzyme B, IFN-gamma, IL-2, IL-4.

***IL-6 measurement by ECLIA method***

IL-6 was measured in 90 patients in the Atezo+Bev group using the ECLIA method. In the 48 cases of the Atezo+Bev training cohort, IL-6 was measured using both methods, and a scatter plot was created based on the IL-6 values obtained from the ECLIA method and the Luminex assay. The conversion formula between the two methods was established using a linear formula. The cutoff value for IL-6 in the validation group, measured using the ECLIA method only, was calculated based on the cutoff value derived from the Luminex assay data in the Atezo+Bev training group, using the above conversion formula　(Supplementary fig. 3).

***Estimation of Immune Cells Infiltration***

For estimating the association of IL-6 expression and tumor immune microenvironment, RNA sequencing data from the liver HCC cohort from The Cancer Genome Atlas Liver Hepatocellular Carcinoma project (TCGA-LIHC) were downloaded from the National Cancer Institute - Genomic Data Commons Data Portal. Subsequently, the gene expression matrix was uploaded to CIBERSORTx (https://cibersortx.stanford.edu/) as a mixture file and run with 1,000 permutations applying the “LM22” signature. As the fraction of 22 types of immune cells sums to one for each sample, it is feasible to compare the fraction of immune cells in different samples.

***Treatment regimen***

For the combination of atezolizumab and bevacizumab, patients received 1200 mg atezolizumab and 15 mg bevacizumab per kg body weight intravenously every 3 weeks. lenvatinib was administered at a dose of 12 mg per day to patients weighing 60 kg or more and at a dose of 8 mg per day to patients weighing less than 60 kg. Patient response to treatment was assessed every 1-3 months using dynamic computed tomography (CT) or magnetic resonance imaging (MRI). Hepatologists and radiologists assessed treatment response according to the modified Response Evaluation Criteria in Solid Tumors (mRECIST) [14] using the following four response categories: complete response (CR), partial response (PR), stable disease (SD), progression of disease (PD). Overall response rate (ORR) was calculated as the sum of patients achieving CR and PR, and disease control rate (DCR) was calculated as the sum of ORR and SD; for DCR and ORR, the best response recorded from the start of treatment to disease progression or relapse was used.

***Statistical Analysis***

Statistical analyses were performed using JMP Pro 17.0.0 (SAS Institute Inc., Cary, NC, USA). Differences between groups were tested using the Mann-Whitney U test for continuous variables and Fisher's exact test for categorical variables. Cochran-Armitage trend tests were used to assess the association between variables with two categories and ordinal variables with k categories. The overall survival (OS) and progression-free survival (PFS) of mRECIST during Atezo+Bev treatment were estimated using the Kaplan-Meier method, and differences between subgroups were assessed using the log-rank test. Factors associated with improvement in PFS were assessed using univariate and multivariate Cox proportional hazards regression models. A p-value < 0.05 and a False Discovery Rate (FDR) < 0.1 was considered to indicate statistical significance.

***References***

1. Zhang P, Ono A, Fujii Y, et al. The presence of vessels encapsulating tumor clusters is associated with an immunosuppressive tumor microenvironment in hepatocellular carcinoma. Int J Cancer. 2022; 151:2278-90.

2. Wu X, Giobbie-Hurder A, Liao X, et al. Angiopoietin-2 as a Biomarker and Target for Immune Checkpoint Therapy. Cancer Immunol Res. 2017; 5:17-28.

3. Zhou W, Zhang X, Feng Y, et al. The CC ligand chemokine family members CCL17/CCL22 predict the survival and response to immune checkpoint blockade therapy of patients with head and neck squamous cell carcinoma. Curr Probl Cancer. 2022; 46:100896.

4. Deng J, Ma X, Ni Y, et al. Identification of CXCL5 expression as a predictive biomarker associated with response and prognosis of immunotherapy in patients with non-small cell lung cancer. Cancer Med. 2022; 11:1787-95.

5. Walsh RM, Ambrose J, Jack JL, et al. Adipose-Tumor Crosstalk contributes to CXCL5 Mediated Immune Evasion in PDAC. bioRxiv. 2023; doi:10.1101/2023.08.15.553432.

6. Tokunaga R, Zhang W, Naseem M, et al. CXCL9, CXCL10, CXCL11/CXCR3 axis for immune activation - A target for novel cancer therapy. Cancer Treat Rev. 2018; 63:40-7.

7. Llovet JM, Castet F, Heikenwalder M, et al. Immunotherapies for hepatocellular carcinoma. Nat Rev Clin Oncol. 2022; 19:151-72.

8. Takada H, Yamashita K, Osawa L, et al. Relationship between Plasma IP-10/CXCL10 Levels and the Initial Therapeutic Response in Patients Treated with Atezolizumab plus Bevacizumab for Unresectable Hepatocellular Carcinoma. Oncology. 2023; 101:655-63.

9. Yang R, Hung MC. The role of T-cell immunoglobulin mucin-3 and its ligand galectin-9 in antitumor immunity and cancer immunotherapy. Sci China Life Sci. 2017; 60:1058-64.

10. Qi Q, Peng Y, Zhu M, et al. Association between serum levels of 12 different cytokines and short-term efficacy of anti-PD-1 monoclonal antibody combined with chemotherapy in advanced gastric cancer. Int Immunopharmacol. 2023; 114:109553.

11. Pasello G, Fabricio ASC, Del Bianco P, et al. Sex-related differences in serum biomarker levels predict the activity and efficacy of immune checkpoint inhibitors in advanced melanoma and non-small cell lung cancer patients. J Transl Med. 2024; 22:242.

12. Chamseddine S, Mohamed YI, Lee SS, et al. Clinical and Prognostic Biomarker Value of Blood-Circulating Inflammatory Cytokines in Hepatocellular Carcinoma. Oncology. 2023; 101:730-7.

13. Ono A, Aikata H, Yamauchi M, et al. Circulating cytokines and angiogenic factors based signature associated with the relative dose intensity during treatment in patients with advanced hepatocellular carcinoma receiving lenvatinib. Ther Adv Med Oncol. 2020; 12:1758835920922051.

14. Lencioni R, Llovet JM. Modified RECIST (mRECIST) assessment for hepatocellular carcinoma. Semin Liver Dis. 2010; 30:52-60.
